# HDACi promotes inflammatory remodeling of the tumor microenvironment to enhance epitope spreading and antitumor immunity

**DOI:** 10.1172/JCI159283

**Published:** 2022-10-03

**Authors:** Andrew Nguyen, Louisa Ho, Richard Hogg, Lan Chen, Scott R. Walsh, Yonghong Wan

**Affiliations:** Department of Pathology and Molecular Medicine, McMaster Immunology Research Centre, McMaster University, Hamilton, Ontario, Canada.

**Keywords:** Immunology, Vaccines, Antigen-presenting cells, Cancer immunotherapy, T cells

## Abstract

Adoptive cell therapy (ACT) with tumor-specific memory T cells has shown increasing efficacy in regressing solid tumors. However, tumor antigen heterogeneity represents a longitudinal challenge for durable clinical responses due to the therapeutic selective pressure for immune escape variants. Here, we demonstrated that delivery of the class I histone deacetylase inhibitor MS-275 promoted sustained tumor regression by synergizing with ACT in a coordinated manner to enhance cellular apoptosis. We found that MS-275 altered the tumor inflammatory landscape to support antitumor immunoactivation through the recruitment and maturation of cross-presenting CD103^+^ and CD8^+^ DCs and depletion of Tregs. Activated endogenous CD8^+^ T cell responses against nontarget tumor antigens were critically required for the prevention of tumor recurrence. Importantly, MS-275 altered the immunodominance hierarchy by directing epitope spreading toward the endogenous retroviral tumor–associated antigen p15E. Our data suggest that MS-275 in combination with ACT multimechanistically enhanced epitope spreading and promoted long-term clearance of solid tumors.

## Graphical abstract

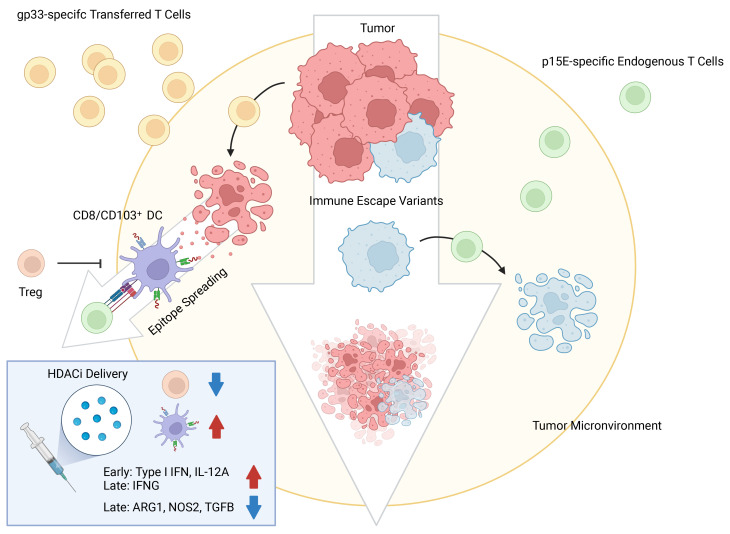

## Introduction

Adoptive cell therapy (ACT) involves the ex vivo expansion and infusion of antigen-specific T cells, tumor-infiltrating lymphocytes (TILs), or engineered T cells expressing T cell receptors (TCRs) or chimeric antigen receptors (CARs) into patients with tumors (1). These personalized cellular products potentiate tumor recognition and killing and has demonstrated clinical efficacy in the treatment of malignant disease (2). However, incomplete responses and tumor recurrence have been reported following adoptive transfer (3). Since patient tumors can harbor extensive variability in antigen expression, targeted therapies such as ACT may create selective pressure for antigen-negative or antigen-low immune escape variants (4). As a result, tumor antigen heterogeneity may reduce the probability of durable responses. It has been demonstrated that the detection of epitope spreading during ACT is highly correlated with improved clinical outcomes (5–7). “Epitope” or “determinant” spreading during cancer immunotherapy is characterized by early tumor lysis and the release of immunogenic tumor-derived antigens (8). Cross-presentation of these antigens by tumor-infiltrating DCs can engage endogenous T cells to facilitate the recognition and killing of a wider tumor antigen repertoire (9). Therefore, deliberate induction of broad-spectrum antitumor immunity during ACT may promote comprehensive eradication of heterogeneous solid tumors.

In opposition, the tumor microenvironment (TME) utilizes an abundant array of immunoregulatory mechanisms that can suppress the activation of endogenous T cell responses. The secretion of immunosuppressive factors by tumors and tumor-infiltrating leukocytes including myeloid-derived suppressor cells (MDSCs), Tregs, and tumor-associated macrophages/neutrophils has been shown to curb the development, survival (e.g., VEGF, IL-6, PGE2), and function (eg. TGF-β, IL-10) of cross-presenting DCs and render them tolerogenic (10, 11). Additionally, the TME impairs the production of proinflammatory DC-activating cytokines (e.g., IFN-α, IFN-β, high mobility group box 1 [HMGB1]), which further inhibits the de novo activation of endogenous T cell responses (12–14). As such, targeting TME immunosuppressiveness may improve epitope spreading and improve the therapeutic impact of ACT. For instance, it has been demonstrated that selective MDSC and Treg depletion (e.g., doxorubicin, paclitaxel (15–17) and targeted molecular inhibition (e.g., indoleamine-pyrrole 2,3-dioxygenase [IDO], STAT3) (18, 19) can foster DC maturation and activity. Alternatively, several methods have been applied to directly potentiate DC function including local upregulation of DC-recruiting cytokines (e.g., GM-CSF, FLT3L) (20–22) and administration of immunostimulatory adjuvants (e.g., TLR, CD40, and stimulator of IFN genes [STING] agonists) (10). Overall, while immunosuppressive pressures within the TME present significant barriers to epitope spreading, strategies to enhance DC activation and cross-presentation may propel the advancement of combinatorial ACT approaches.

In this study, we determined that MS-275, a class I histone deacetylase inhibitor (HDACi), delivered in conjunction with ACT could promote sustained tumor regression and prevent relapse. This was associated with inflammatory remodeling of the TME in favor of recruitment and activation of cross-presenting DCs. Decreased immunosuppressive signaling also corresponded with a depletion of tumor-infiltrating Tregs. These changes collectively promoted epitope spreading to nontarget tumor antigens that were critical for long-term tumor control. Overall, MS-275 multi-mechanistically improved epitope spreading to enhance the efficacy of ACT.

## Results

### MS-275 delivery enhances ACT, leading to sustained tumor regression.

During ACT, the in vivo magnitude and persistence of infused tumor-specific T cells are considered determinants of successful clinical responses (23). It has been increasingly accepted that less differentiated subsets of memory T cells contribute to durable antitumor immune responses with exceptional proliferative capacity upon antigenic stimulation (24, 25).

**Figure 1:**
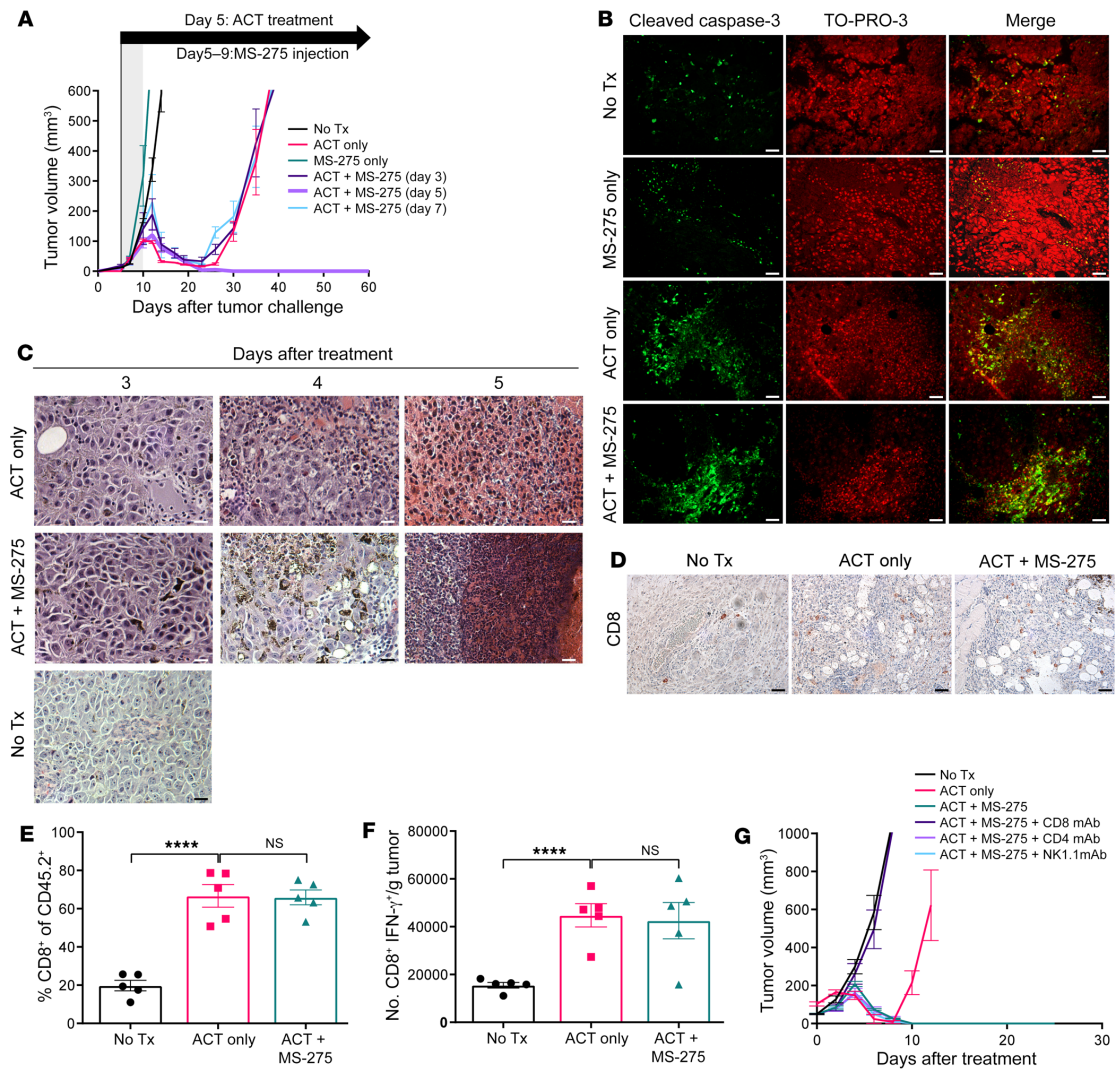
Concomitant MS-275 delivery prevents tumor relapse during ACT. In C57BL/6 mice (*n* = 5 per group), (**A**) 5-day-old intradermal B16-gp33 tumors were treated with ACT (10^4^ LCMV GP_33–41_–specific Tmem cells delivered i.v. followed by viral vaccination). MS-275 was injected i.p. daily for 5 days starting at various time points. Tumor volumes were calculated on the basis of tumor height, width, and length. (**B**) IF staining for anti–cleaved caspase 3 antibody and TO-PRO-3 nuclear staining (scale bars: 25 μm), (**C**) H&E staining (scale bars: 10 μm), and (**D**) IHC anti-CD8 antibody staining (scale bars: 25 μm) of frozen tumor sections harvested 5 days after treatment. Digested tumors (*n* = 5 per group) were enriched for CD45.2^+^ cells, and the (**E**) frequency of CD45.2^+^CD8^+^ T cells and (**F**) absolute count of LCMV GP_33–41_–specific CD8^+^ T cells as determined by IFN-γ expression after ex vivo peptide stimulation were measured. (**G**) Tumor volume measurements following selective lymphocyte depletion prior to and during ACT+MS-275 treatment using mAbs specific for CD8, CD4, and NK1.1. Data are presented as the mean ± SEM . ****P < 0.0001, by 1-way ANOVA. No Tx, no treatment.

To generate LCMV GP_33–41_–specific memory T (Tmem) cells for adoptive transfer, we infected C57BL/6 mice with lymphocytic choriomeningitis virus (LCMV, Armstrong strain) and harvested bulk splenocytes after 1 month. In mice intradermally challenged with B16-F10 murine melanoma cells expressing the immunodominant LCMV GP_33–41_ epitope (B16-gp33), ACT treatment utilizing Tmem cells was sufficient to induce complete regression of 5-day-old tumors; however, the tumors quickly relapsed within 1 month after initial tumor challenge. The class I HDACi MS-275 has been previously shown to potentiate immunotherapeutic outcomes (26, 27), so we speculated whether incorporating the drug could prolong tumor regression. Daily injections of MS-275 were delivered concomitantly with ACT (day 5) or 2 days prior/later (day 3, day 7) and continued for 4 additional days. While MS-275 alone did not provide tumor protection, ACT plus MS-275 (ACT+MS-275) completely sustained tumor regression relative to ACT alone (Figure 1A). Interestingly, early or late delivery of MS-275 abrogated any therapeutic benefit, signifying that timing-dependent interactions between ACT and MS-275 were necessary to prevent tumor relapse.

Since MS-275 has anticancer properties (28, 29), we considered whether the drug was additively contributing to tumor regression by direct elimination of resistant tumor variants despite being unable to control tumor growth on its own. In frozen tumor sections obtained 5 days after treatment, immunofluorescence staining for cleaved caspase 3 revealed that MS-275 alone did not seem directly promote tumor apoptosis (Figure 1B). By contrast, ACT+MS-275 treatment demonstrated drastically higher levels of cleaved caspase 3 relative to ACT alone, suggesting that MS-275 synergized with ACT to drive tumor apoptosis.

H&E staining of tumor tissue revealed that ACT-treated tumors were heavily infiltrated with leukocytes in both the presence and absence of MS-275 (Figure 1C). To determine whether MS-275 enhances ACT by selectively increasing the total number of tumor-infiltrating CD8^+^ T cells, we stained tumor sections with anti-CD8 antibody for immunohistochemical analysis 5 days after treatment. We observed no significant difference in total CD8 staining with the addition of MS-275 (Figure 1D), which was further validated by flow cytometric analysis (Figure 1E). Furthermore, the total number of LCMV GP_33–41_–specific CD8^+^ T cells was not affected by MS-275 (Figure 1F). Although MS-275 could mobilize other lymphocytes with antitumor potential, selective depletion of CD4 or NK1.1-expressing cells during ACT+MS-275 treatment provided no loss of sustained tumor regression compared with CD8 depletion (Figure 1G). Taken together, despite the fact that tumor control was primarily CD8^+^ T cell dependent, the additional therapeutic benefit afforded by concomitant MS-275 delivery may not have relied on the additional recruitment of target antigen–specific CD8^+^ T cells or other antitumor lymphocyte populations.

### Inflammatory remodeling of the TME favors immunoactivation.

Since the data suggest that ACT-MS-275 synergy occurs within a restricted time frame, we began to investigate the dynamic drug-induced changes within the TME that may facilitate enhanced tumor killing. To accomplish this, we conducted microarray analyses (Gene Expression Omnibus [GEO]GSE179337) of bulk tumor RNA from ACT-treated mice with or without MS-275 at 1, 3, and 5 days after treatment.

Using connectivity mapping (CMap), we first confirmed whether the gene changes observed were due to the direct influence of MS-275. We performed time-course analysis by measuring differential gene expression between ACT with or without MS-275 from day 1 to day 5 after treatment. Lists of differentially expressed genes (DEGs) that showed a greater than 1.5 or a greater than 2.0 absolute fold change (FC) were compared with the expression data from chemical perturbation studies of thousands of compounds within the CMap library. Of the 32 chemicals that showed significant overlap in differential gene expression, chemical studies utilizing MS-275 had the highest similarity to our expression data (Supplemental Figure 1; supplemental material available online with this article; https://doi.org/10.1172/JCI159283DS1). As a result, the data suggested that concomitant MS-275 delivery during ACT treatment altered the local genetic landscape through direct chemical perturbation and imposed a unique signature that was not easily reproduced by other drugs.

**Figure 2:**
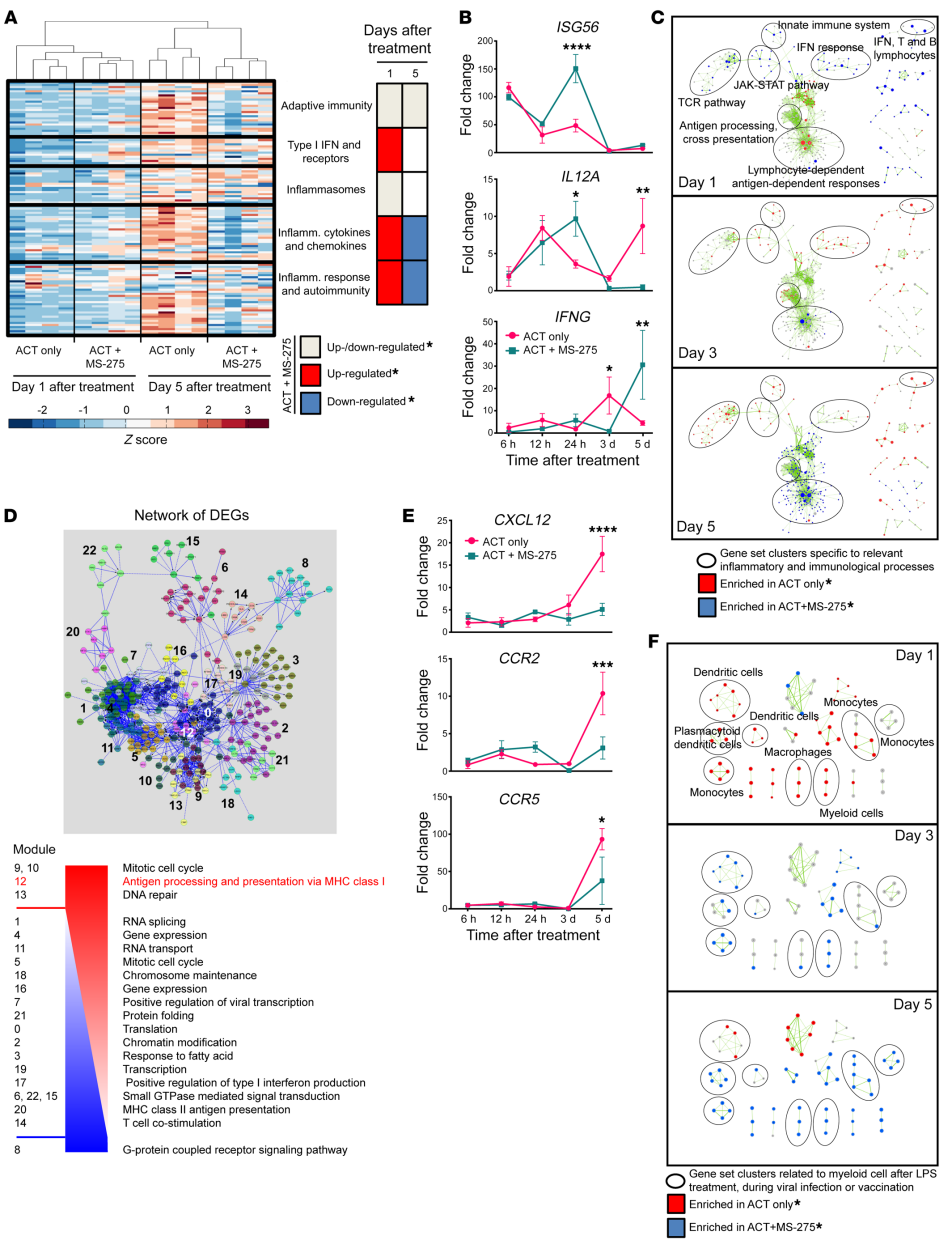
MS-275 remodels the inflammatory landscape of the TME to promote antigen processing and presentation. Bulk tumor RNA was derived from ACT-treated mice with or without MS-275 for microarray analysis (*n* = 4 per group). (**A**) Heatmap and GSEA of custom gene sets representing specific inflammatory pathways on day 1 and day 5 (see also Supplemental Table 2). (**B**) qRT-PCR of proinflammatory cytokines at specific time points after treatment (*n* = 3 per group). (**C**) GSEA of curated gene sets (C2) derived from the MSigDB and displayed as an enrichment map (see also Supplemental Figure 2). (**D**) GO analysis of modules (see also Supplemental Table 3) derived from DEGs (day 3 vs. day 1 and day 5 vs. day 1; FDR *P* < 0.05) within a PPI network. Modules were sorted by the ratio of upregulated to downregulated genes when comparing ACT+MS-275 with ACT alone (see also Supplemental Figure 3). (**E**) qRT-PCR of myeloid-related chemokines at specific time points after treatment (*n* = 3 per group). (**F**) GSEA of immunologic signatures (C7) derived from the MSigDB, where highlighted groups represent gene sets related to activated myeloid cells (see also Supplemental Figure 4). *FDR *P* < 0.05 (**A**, **C**, and **F**). Data are presented as the mean ± SEM. *P < 0.05, **P < 0.01, ***P < 0.001, and ****P < 0.0001, by unpaired Student’s *t* test (**B** and **E**).

To assess the tumor inflammatory state over the course of treatment, we analyzed custom gene sets (Supplemental Table 1) representing specific inflammatory pathways for statistical enrichment within the expression data for each treatment group (Figure 2A and Supplemental Table 2). Relative to ACT alone, ACT+MS-275 upregulated type I IFN signaling, inflammatory cytokine signaling, and inflammatory responses at an early time point (day 1), while downregulating those processes at a later time point (day 5). Despite having a progressive antiinflammatory effect, quantitative reverse transcription PCR (qRT-PCR) validation showed that ACT+MS-275 also enhanced the expression of select proinflammatory cytokines at various time points. In particular, MS-275 upregulated *ISG56* and *IL-12* expression on day 1 and *IFNG* expression on day 5 (Figure 2B and Supplemental Figure 2). Taken together, MS-275–driven modulation of tumor inflammation may activate biological pathways that can facilitate sustained tumor regression.

We then used gene set enrichment analysis (GSEA) to interrogate the effect of MS-275 on biological pathways derived from curated gene sets (C2) in the Molecular Signatures Database (MSigDB) (Figure 2C). An enrichment map was constructed to group significantly enriched gene sets containing similar genes. On the basis of the differential enrichment pattern of clustered gene sets, we noted that ACT+MS-275 treatment promoted early upregulation (day 1) of TCR signaling, JAK/STAT signaling, and innate immunity followed by late upregulation (day 5) of antigen processing, cross-presentation, and lymphocyte-dependent, antigen-dependent responses. Since ACT-only treatment showed an inverse enrichment pattern with the aforementioned biological processes, the data confirmed that MS-275–induced inflammatory changes correlated with enhanced immunoactivation.

Gene ontology (GO) analysis allowed us to determine whether terms representing annotated biological processes were statistically overrepresented in our DEGs and comparable to our GSEA-enriched MSigDB pathways. Using DEGs obtained from comparing ACT+MS-275 versus ACT alone over a time course (day 3 vs. day 1 and day 5 vs. day 1), a protein-protein interaction (PPI) network was constructed, and gene modules were identified from the network (Supplemental Table 3). GO analysis allowed us to functionally classify the modules according to annotated biological processes. After ordering the modules by the percentage of constituent genes upregulated by MS-275, we observed that antigen processing and cross-presentation was highly upregulated throughout the time course (Figure 2D and Supplemental Figure 3).

Since immunoactivation may represent a crucial mechanism for promoting sustained tumor regression, we hypothesized that the effect of MS-275 on tumor inflammation may alter the local composition of tumor-infiltrating myeloid cells. Various myeloid chemotactic genes such as *CCR5*, *CCR2*, and *CXCL12* have been associated with tolerogenic DC/MDSC infiltration and the immunosuppression of antitumor responses (30–32). qRT-PCR analysis showed that MS-275 potently downregulated the expression of these chemokines by day 5, which may have influenced the recruitment of immunoactivating myeloid cells to the tumor (Figure 2E). Indeed, GSEA of immunologic signatures (C7) from the MSigDB revealed that gene sets related to activated myeloid cells from LPS treatment, viral infection, or vaccination were enriched by ACT+MS-275 treatment from day 1 to day 5 (Figure 2F and Supplemental Figure 4). Taken together, examination of tumor-infiltrating myeloid cells is necessary to determine how MS-275 promotes enhanced antitumor immunity.

### MS-275 drives myeloid cell recomposition and maturation within the tumor and draining lymph node.

Myeloid cells are a heterogenous group of innate immune cells that exist in varying activation and differentiation states (33, 34). Tumors exploit myeloid cell plasticity through the secretion of soluble factors that can divert myelopoiesis and skew myeloid cell function to support tumor growth (35). In the context of ACT, we questioned whether MS-275 could recompose the tumor-infiltrating myeloid cell compartment into one with effective antigen-presenting cells with CD8^+^ T cell–activating capacity.

**Figure 3:**
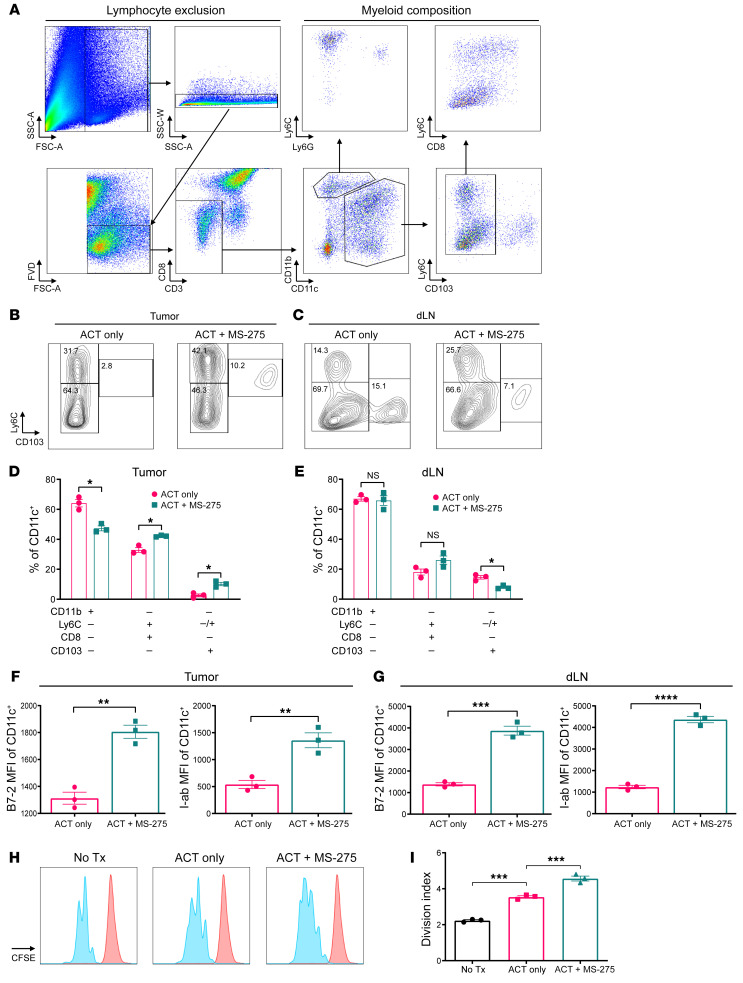
MS-275 alters myeloid cell composition within the tumor and dLN and enhances costimulation. Five days after treatment, digested tumors (*n* = 3 per group) were positively enriched for CD45.2 cells. (**A**) Representative scatter plots outlining the gating strategy for characterizing myeloid cell populations in the tumor and dLNs. SSC-A, side scatter area; FSC-A, forward scatter area. Myeloid cell composition changes in the tumor and dLNs during ACT with or without MS-275 treatment are depicted by (**B** and **C**) representative contour plots and (**D** and **E**) frequency as a percentage of total CD11c^+^ cells. Maturation marker expression levels in total CD11c^+^ cells in the (**F**) tumor and (**G**) dLN were determined by MHC class II– (I-Ab) and costimulatory ligand CD86–specific (B7-2–specific) flow staining. (**H** and **I**) Enriched CD11c^+^ cells were pulsed with LCMV GP_33–41_ peptide and cocultured with CFSE-labeled LCMV-P14 TCR-transgenic naive T cells. Representative histograms show CFSE dilution after 3 days, and changes in proliferation due to treatment were quantified using the division index. Data are presented as the mean ± SEM. *P < 0.05, ** *P* < 0.01, *** *P* < 0.001, and ****P < 0.0001, by Student’s *t* test (**D**–**G**) or 2-way ANOVA (**I**).

Five days after treatment with ACT, with or without MS-275, we characterized myeloid cell populations in the tumor and draining lymph nodes (dLNs) by flow cytometry. After excluding lymphocytes, we identified several cell subsets including monocytic (CD11c^–^CD11b^+^Ly6C^hi^Ly6G^–^), granulocytic (CD11c^–^CD11b^+^Ly6C^lo^Ly6G^–^), as well as dendritic (CD11c^+^CD11b^+^Ly6C^–^CD8^–^/CD11c^+^ CD11b^–^Ly6C^+^CD8^+^/CD11c^+^CD11b^–^CD8^–^CD103^+^) cell populations (Figure 3A). While we had previously reported MS-275’s influence on tumor-infiltrating monocytic/granulocytic myeloid cells (36), we expanded our analysis to include DCs and observed cellular subset recomposition within the tumor and dLNs (Figure 3, B and C). In the tumor, ACT+MS-275 treatment reduced the frequency of CD11b^+^ DCs, while increasing the frequency of CD8^+^ and CD103^+^ DCs (Figure 3D); in dLNs however, we detected a reduced frequency of CD103^+^ DCs (Figure 3E). In addition, DCs in the tumor and dLNs demonstrated a significant increase in maturation marker expression including expression of MHC class II (I-Ab) and costimulatory ligand CD86 (B7-2) (Figure 3, F and G). This coincided with higher immunoactivation potential when we pulsed the DCs with LCMV GP_33–41_ peptide and cocultured them with CFSE-labeled LCMV-P14 TCR-transgenic naive T cells ex vivo (Figure 3, H and I). Overall, MS-275–dependent tumor remodeling may mobilize immunoactivating DCs to promote endogenous CD8^+^ T cell responses.

### Activation of endogenous CD8^+^ T cell responses promotes sustained tumor regression.

**Figure 4:**
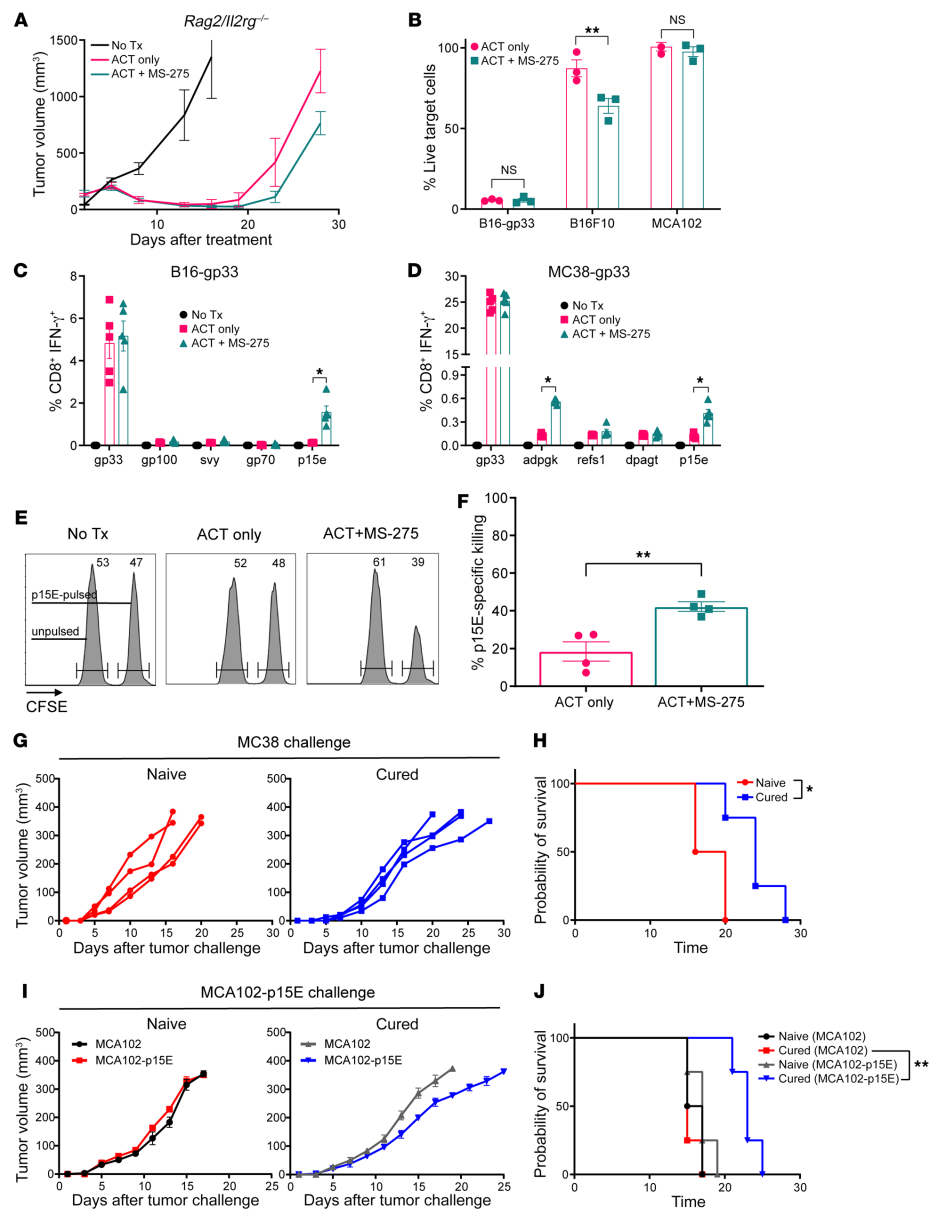
Epitope spreading mobilizes tumor-rejecting, p15E-specific endogenous CD8^+^ T cell responses. (**A**) In lymphocyte-deficient *Rag2/Il2rg*-DKO mice (*n* = 7–8 per group), 5-day-old intradermal B16-gp33 tumors were treated with ACT with or without MS-275. Tumor volumes were calculated on the basis of height, width, and length. Using C57BL/6 tumor-bearing mice treated with ACT+MS-275, (**B**) CD8^+^ T cells were positively enriched from digested tumors and cocultured with target cell lines at a 10:1 effector to target ratio. Killing was measured by MTT reduction and done in triplicate. (**C** and **D**) Five days after vaccination, the frequency of tumor antigen–specific CD8^+^ T cells in peripheral blood was determined by IFN-γ expression after ex vivo stimulation with peptides (*n* = 3 per group). (**E** and **F**) CFSE^hi^-labeled bulk splenocytes were pulsed with p15E peptide, mixed with CFSE^lo^-labeled, unpulsed splenocytes at a 1:1 ratio, and infused into tumor-bearing mice 5 days after ACT+MS-275 treatment. P15E-specific killing was measured by the recovery of labeled, pulsed targets relative to unpulsed targets (*n* = 4 per group). Mice that were cured of B16-gp33 tumors during ACT+MS-275 were rechallenged with (**G** and **H**) natural, p15E-expressing MC38 tumors or (**I** and **J**) engineered, p15E-overexpressing MCA102 tumors and monitored for tumor growth and survival (*n* = 4 per group). Data are presented as the mean ± SEM. *P < 0.05 and **P < 0.01, by unpaired Student’s *t* test (**B** and **F**), 1-way ANOVA (**C** and **D**), or log-rank test (**H** and **J**).

To confirm that endogenous CD8^+^ T cells enhanced the therapeutic efficacy of ACT treatment, lymphocyte-deficient *Rag2/Il2rg* double-knockout (*Rag2/Il2rg*-DKO) mice were treated with ACT+MS-275. We observed a failure to recapitulate the therapeutic effects of MS-275, resulting in tumor control similar to that seen with ACT alone (Figure 4A). The data therefore suggest that endogenous CD8^+^ T cells were necessary for tumor clearance.

As demonstrated previously, the magnitude of LCMV GP_33–41_–specific CD8^+^ T cell responses remained unchanged during MS-275 delivery. If cross-presentation of tumor peptides during ACT enhanced endogenous CD8^+^ T cell responses against targets other than LCMV GP_33–41_, then broadening the spectrum of antitumor killing may represent an important mechanism to prolong tumor regression. To confirm that MS-275–potentiated tumor killing transcended LCMV GP_33–41_ epitope recognition, we investigated whether tumor-infiltrating endogenous CD8^+^ T cells derived from ACT+MS-275–treated mice could recognize and kill parental B16F10 cells, which do not express LCMV GP_33–41_. Using a 10:1 effector/target coculture approach, we did not observe improvements in B16-gp33 lysis, but there was a significant increase in B16F10 cell death (Figure 4B). Moreover, the inclusion of MCA102 fibrosarcoma cells as an irrelevant line suggested that the enhanced tumor killing was antigen dependent.

Using a limited epitope screening process, we established the antigen specificity of endogenous CD8^+^ T cell responses during ACT+MS-275 treatment. In B16-gp33 tumors, we observed an increased frequency of CD8^+^ T cells specific for p15E, an endogenous retroviral tumor-associated antigen (Figure 4C). Interestingly, T cell responses against defined melanoma antigens (gp100, svy) and other retroviral gene products (gp70) were unchanged. To confirm whether epitope spreading altered the immunodominance hierarchy to disproportionally favor p15E-specific responses, we repeated the experiment with the MC38 colon adenocarcinoma tumor model, which expresses p15E. In MC38 tumors expressing LCMV GP_33–41_ (MC38-gp33), MS-275 raised the frequency of T cell responses to p15E as well as to a neoepitope corresponding to a mutation in ADP-dependent glucokinase (Adpgk) (Figure 4D). Although the extent of epitope spreading may be model dependent, we demonstrated that MS-275 could promote endogenous immune responses against nontarget tumor antigens.

However, it was yet unclear whether these responses translated to in vivo antigen–specific killing. We pulsed CFSE-labeled bulk splenocytes with p15E peptide and infused them into B16-gp33 tumor–bearing mice 5 days after ACT+MS-275 treatment. Interestingly, we observed that enhanced p15E responses correlated with improved killing of p15E-pulsed target cells in vivo (Figure 4, E and F). Since MC38 tumor cells share an endogenous antigen with B16F10 in the expression of p15E (37, 38), we wondered whether ACT+MS-275–driven p15E responses alone could facilitate tumor rejection. In mice that were cured of B16-gp33 tumors during ACT+MS-275 treatment, rechallenge with parental MC38 tumors resulted in delayed tumor growth and longer survival compared with naive mice (Figure 4, G and H). This suggested that persisting immunity from ACT+MS-275 treatment could have mild protective benefits against tumors expressing p15E. To eliminate cell line differences, we repeated the experiment but rechallenged cured mice with parental MCA102 cells (inherently p15E-negative) or MCA102 cells engineered to express p15E (MCA102-p15E). Again, we demonstrated that MCA102-p15E cells showed delayed tumor growth in cured mice relative to parental MCA102 cells (Figure 4, I and J). Ultimately, MS-275–dependent immunoactivation of endogenous CD8^+^ T cells allow for tumor rejection beyond target antigen recognition.

### Intratumoral downregulation of immunosuppressive signals coincide with Treg depletion.

Tregs have indispensable functions in inducing and maintaining self-tolerance and immune homeostasis, and their presence is often associated with a poor clinical prognosis in cancer (39). Many studies support a mutualistic relationship between immunosuppressive tumor-infiltrating myeloid cells and Tregs. Myeloid cells can secrete IL-10 and TGF-β, whereas Tregs secrete IL-4, IL-13, and IL-10, which results in reciprocal activation (40). Furthermore, nitric oxide synthase and arginase production by myeloid cells has been found to be a potent inducer of Treg activity (41–43). Since MS-275 remodels the TME and facilitates myeloid cell recomposition and activation, we questioned whether that would create a subversive effect on tumor-infiltrating Tregs.

**Figure 5:**
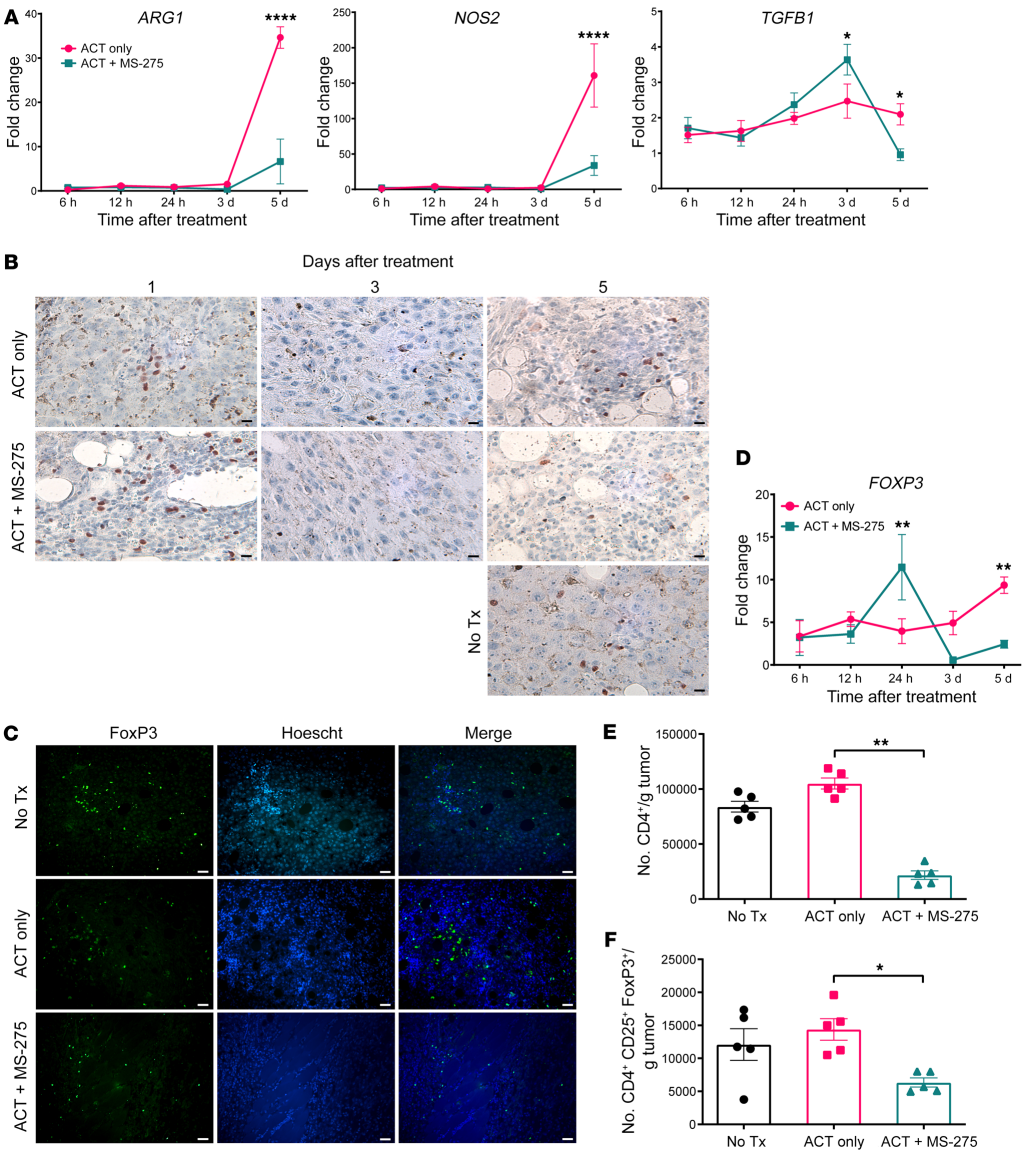
Reduced local immunosuppressive signals coincide with reduced Treg infiltration. (**A** and **D**) Bulk tumor RNA was derived from mice subjected to ACT with or without MS-275, and qRT-PCR analysis was performed at specific time points after treatment (*n* = 3 per group). (**B** and **C**) Five days after treatment, frozen tumor sections were stained for anti-Foxp3 antibody in IHC (scale bars: 10 μm) and IF (scale bars: 25 μm) imaging experiments. (**E** and **F**) Positive enrichment of CD45.2^+^ cells from digested tumors in treated mice was followed by flow cytometric staining, and absolute Treg counts were measured (*n* = 5 per group). Data are presented as the mean ± SEM. *P < 0.05, **P < 0.01, and ****P < 0.0001, by unpaired Student’s *t* test (**A** and **D**) or 1-way ANOVA (**E** and **F**).

qRT-PCR analysis of bulk tumor RNA indicated that ACT+MS-275 treatment downregulated *ARG1*, *NOS2*, and *TGFB1* expression, which may prohibit Treg expansion (Figure 5A). To examine the corresponding impact of ACT+MS-275 treatment on Treg abundance, we stained frozen tumor sections with anti-Foxp3 antibody for immunohistochemistry and immunofluorescence imaging. While ACT alone increased Treg numbers relative to those in untreated mice, ACT+MS-275 increased Tregs initially (day 1), but decreased their number substantially afterwards (days 3–5) (Figure 5, B and C). These trends were validated by qRT-PCR analysis of *FOXP3* gene expression in bulk tumor RNA (Figure 5D). Flow cytometric staining of CD45.2-enriched tumor-derived cells further revealed that depletion of tumor-infiltrating CD4^+^CD25^+^Foxp3^+^ Tregs was accompanied by a more general and severe depletion of tumor-infiltrating CD4^+^ T cells (Figure 5, E and F).

### Tregs inhibit endogenous CD8^+^ T cell responses and prevent long-term tumor control.

Tregs can induce immunosuppression through a variety of mechanisms including secretion of immunosuppressive cytokines, competitive consumption of IL-2, direct killing via perforin and granzyme pathways, and direct subversion of antigen-presenting cell function through downregulation of costimulatory molecules (44). To evaluate the therapeutic contribution of Treg ablation in the context of ACT, we depleted CD4^+^ T cells using mAbs or used “depletion of regulatory T cell” (DEREG) BAC-transgenic mice, which express a simian diphtheria toxin receptor–enhanced green fluorescent protein (DTR-EGFP) fusion protein under control of the endogenous FoxP3 promoter/enhancer regions on the BAC transgene (45), to ablate Tregs via i.p. diphtheria toxin (DT) administration.

**Figure 6:**
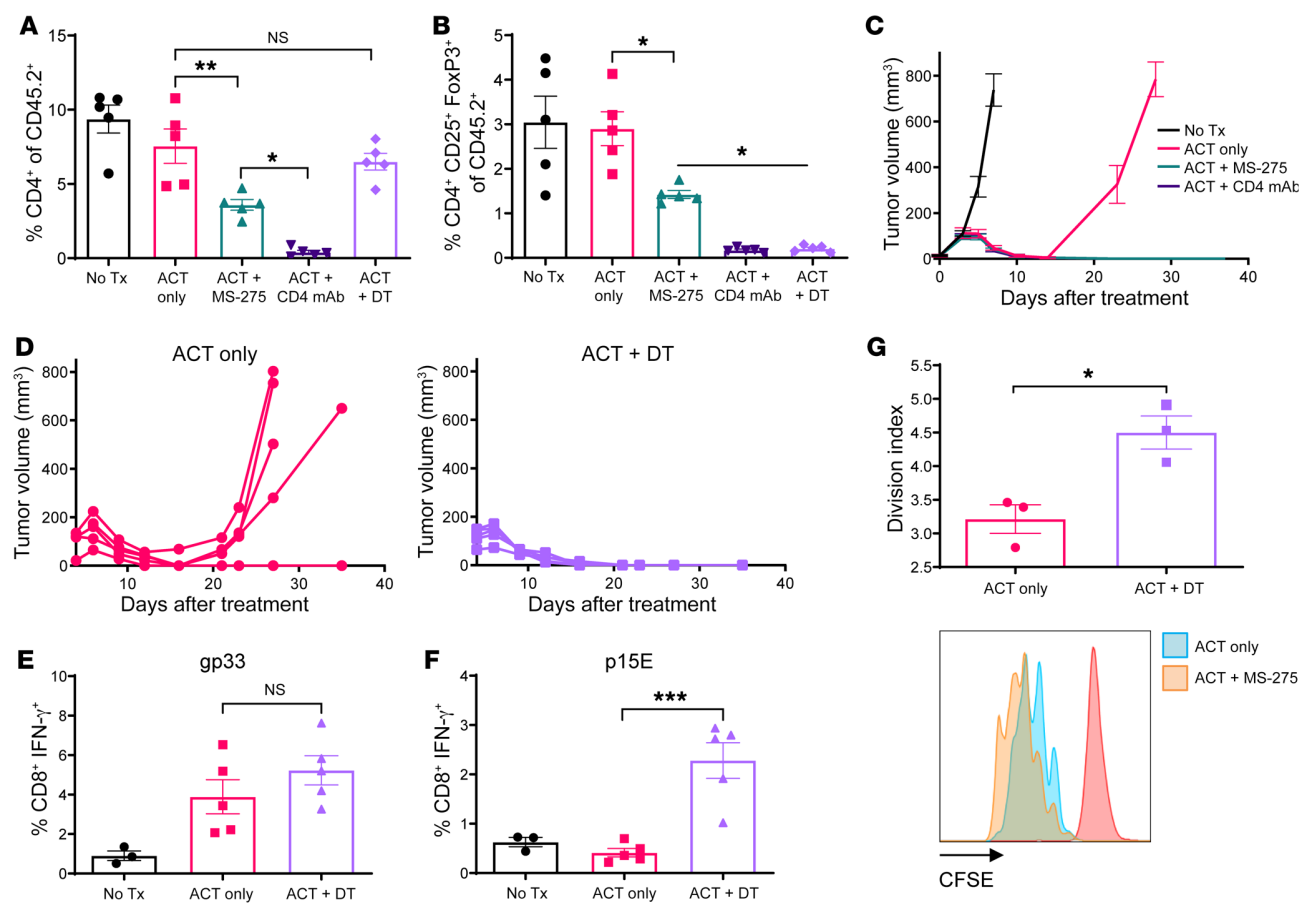
Tregs selectively obstruct the magnitude of endogenous responses from epitope spreading, and their depletion promotes sustained tumor regression. In DEREG BAC-transgenic mice (*n* = 5 per group), 5-day-old intradermal B16-gp33 tumors were treated with ACT followed by depletion of Tregs using anti-CD4 mAbs or DT. (**A** and **B**) Five days after treatment, Tregs were quantified within peripheral blood by flow cytometry. (**C** and **D**) Tumor volume measurements following Treg depletion in the context of ACT. (**E** and **F**) Five days after vaccination, the frequency of tumor antigen–specific CD8^+^ T cells in peripheral blood was determined by IFN-γ expression after ex vivo stimulation with peptides. (**G**) Five days after treatment, digested tumors (*n* = 3 per group) were positively enriched for CD11c^+^ cells, pulsed with LCMV GP_33–41_ peptide, and cocultured with CFSE-labeled, LCMV-P14 TCR-transgenic naive T cells. Representative histograms show CFSE dilution after 3 days relative to the unstimulated control (red peak), and changes in proliferation due to treatment were quantified using the division index. Data are presented as the mean ± SEM . *P < 0.05, **P < 0.01, and ***P < 0.001, by 1-way ANOVA (**A**, **B**, **E**, and **F**) or unpaired Student’s *t* test (**G**).

Flow cytometric analysis of peripheral blood 5 days after treatment revealed that, whereas MS-275 induced partial depletion of bulk CD4^+^ T cells and CD4^+^CD25^+^Foxp3^+^ Tregs, CD4^+^ mAb delivery completely depleted both subsets (Figure 6, A and B). Comparatively, DT administration preserved the bulk CD4^+^ T cell compartment while completely ablating CD4^+^CD25^+^Foxp3^+^ Tregs. In either scenario, the therapeutic benefit of MS-275 was recapitulated, and sustained tumor regression was achieved (Figure 6, C and D). Therefore, the influence of MS-275 on Treg numbers may play a critical role in its therapeutic efficacy. Interestingly, DT administration increased the frequency of p15E-specific CD8^+^ T cells but had a negligible impact on LCMV GP_33–41_–specific CD8^+^ T cells (Figure 6, E and F), suggesting that Tregs selectively inhibit the magnitude of endogenous CD8^+^ T cell responses to facilitate tumor relapse. When CD11c^+^ cells were isolated from ACT+DT-treated tumors and cocultured with P14-naive T cells, we observed greater proliferation compared with ACT alone (Figure 6G). Taken together, our findings suggest that MS-275 may have instigated concordant mechanisms of enhanced immunoactivation to promote sustained tumor regression via myeloid cell recomposition and Treg depletion.

## Discussion

The density and composition of tumor-infiltrating immune cells often predict the efficacy of immunotherapy. Broadly speaking, “cold tumors” are characterized by low proinflammatory cytokine production, T cell infiltration, and molecular signatures of immune activation (46). Furthermore, cold tumors orchestrate the poor cellular fate of tumor-infiltrating lymphocytes by cultivating an immunosuppressive microenvironment. Reciprocal host-tumor interactions lead to the propagation of local antiinflammatory signals and an influx of immunosuppressive cells. In particular, MDSCs, Tregs, tumor-associated DCs (TADCs), and type 2–polarized macrophages (M2s) are intrinsically associated with the developing TME and coordinate antiinflammatory mechanisms to obstruct the function of cytotoxic CD8^+^ T cells and antigen-presenting cells (47). As a result, strategies that promote tumor inflammation (“hot tumors”) may circumvent TME immunosuppressive phenotypes and enhance the efficacy of T cell immunotherapy.

With our ACT platform, we were able to observe significant T cell infiltration into solid tumors and complete acute regression. ACT alone also induced higher local inflammatory signaling relative to ACT+MS-275, suggesting hot tumor induction; however, this corresponded with worse immunologic and clinical outcomes, including reduced antigen presentation and antigen-dependent responses and tumor relapse. Indeed, tumor inflammation does not always predispose the TME toward immunostimulation and can instead promote immune escape (48). It has been demonstrated that tumor exposure to IFN-γ upregulates PD-L1 expression, such that subsequent engagement to programmed cell death–expressing (1 PD-1–expressing) T cells attenuates their antitumor response (49). Similarly, programmed death ligand 1 (PD-L1) upregulation on CD103^+^ DCs from tumor dLNs impaired cross-presentation, whereas PD-L1 and PD-1 blockade mitigated DC dysfunction (50). Finally, inflammation has been shown to drive the accumulation of MDSCs and Tregs (51), enhancing tumor immunosuppressive effects on infiltrating immune cells. Attenuating excessive inflammation in hot tumors may therefore allow immunotherapeutic strategies to have a durable clinical outcome.

Histone acetylation can alter chromatin structure and gene transcription to affect various aspects of the TME including tumor immunogenicity, T cell infiltration, and immunosuppression (52). In the context of ACT, we demonstrated that the class I HDACi MS-275 promotes sustained tumor regression. Despite having a broadly antiinflammatory effect over time relative to ACT-only treatment, ACT+MS-275 enhanced gene signatures related to immunoactivation. We speculate that MS-275 achieves this by altering the composition and dynamics of intratumoral inflammatory signaling with emphasis on early upregulation of type I IFN signaling and late upregulation of type II IFN signaling. IFN-α has been shown to enhance DC maturation and cross-presentation through antigen survival, endocytic routing, and processing (53). In support of this, we detected increased *ISG56* and *IL12* expression (mature DC–derived cytokines) within 24 hours of treatment that coincided with an accumulation of inflammatory myeloid gene signatures over time. As previously stated, IFN-γ may have deleterious effects on antigen presentation and can potentiate immune escape. During ACT+MS-275 treatment, *IFNG* expression within the tumor peaked 5 days after treatment, coinciding with peak systemic T cell responses. ACT-only treatment produced peak *IFNG* expression on day 3, suggesting that earlier exposure to IFN-γ may predispose mice to eventual therapeutic failure. Ultimately, if coordinated expression of select inflammatory signals can curtail tumor immunosuppression and maximize antitumor immunity, MS-275 may promote features of what we term a “warm tumor.”

In the TME, the acquisition, processing, and cross-presentation of extracellular tumor antigen released from dying tumor cells by tumor-infiltrating DCs is critical for antitumor immunity. Mouse conventional DCs (cDCs) comprise 2 main subsets, CD8^+^ or CD103^+^ cDC1 subsets and CD11b^+^ cDC2 subsets. cDC1s are often associated with superior antigen cross-presentation, stronger CD8^+^ T cell immunity, and improved clinical prognosis (10). However, tumors often subvert the maturation and function of infiltrating cDCs, such that they become protumorigenic and suppress immune activation. These tolerogenic DCs present tumor antigen without proper costimulation (CD80/CD86), express inhibitory molecules (PDL1/CTLA4), and secrete immunosuppressive factors (TGF-β, IL-10, IL-27, NO, Arg, and IDO) (54). Depletion of tolerogenic DCs has been associated with reduced tumor growth and angiogenesis (55, 56), and, as such, targeting these cells may improve the recruitment, infiltration, and effector activity of T cells in the TME. During ACT+MS-275 treatment, we observed that intratumoral changes to the chemokine milieu were associated with cDC subset recomposition in the tumor/dLN, favoring CD8^+^ and CD103^+^ cDC1 accumulation within the TME. Additionally, MS-275–induced tumor inflammation was accompanied by an increase in costimulatory molecule expression (CD86/I-Ab) and antigen-presenting capability. Overall, MS-275 simultaneously subverted tolerogenic DC activity and promoted immunoactivation by recomposing the TME and providing maturation signals to support tumor-infiltrating cDC1s.

Cross-presentation of tumor antigens by cDCs is a prerequisite to epitope spreading. In cancer immunotherapy, this process leads to the enhancement and diversification of endogenous T cell responses against different epitopes from the original target. In the context of ACT and other immunotherapies, epitope spreading was observed in patients achieving remission of metastatic lesions and may thus contribute to treatment responsiveness (57). However, the association between epitope spreading and clinical benefit has been mostly correlative. It is still unclear if and how therapeutic success is mechanistically dependent on epitope spreading. It has been suggested that epitope spreading can eliminate emergent immune escape variants as a result of therapeutic selective pressure (58). Alternatively, it may prolong antitumor immunity by stimulating endogenous T cell responses that can persist after the contraction of the initial therapy (59). During ACT+MS-275 treatment, we demonstrated that endogenous CD8^+^ T cells were critical for preventing tumor relapse and that selective enhancement of p15E-specific responses provided long-lived recognition and killing of p15E-expressing tumors. Epitope spreading was therefore critical for the prevention of tumor relapse during ACT.

Tregs exert indispensable functions in inducing and maintaining self-tolerance and immune homeostasis. During cancer, Tregs infiltrate into tumor tissue, and their presence is associated with poor clinical prognosis (39, 60). Correspondingly, the systemic removal of Tregs can invoke effective antitumor immunity (61, 62). In this study, we observed that MS-275 directly affected the TME and/or recomposed the myeloid department to reduce intratumoral production of TGF-β, NO, and Arg, leading to partial depletion of Tregs. This provided direct therapeutic value, since complete ablation of Tregs during ACT was able to prevent tumor relapse in lieu of MS-275 treatment. Moreover, Treg ablation did not impact LCMV GP_33–41_–specific T cell responses but markedly upregulated p15E-specific responses, suggesting that Treg accumulation in tumors selectively inhibits epitope spreading responses.

## Methods

### Study design.

The overall objective of the study was to determine how epigenetic modification during ACT can prevent tumor recurrence. The in vivo experiments were conducted to examine the differential therapeutic effect of HDACi delivery, to characterize local genetic, inflammatory, and immunological changes, and to determine the therapeutic impact of epitope spreading in the context of tumor recurrence prevention. Studies were performed with 6-week-old female age-matched mice (in vivo, *n* = 5 per group; ex vivo, *n* = 3 per group). Experiments were repeated at least 3 times. Tumor-challenged mice were randomized prior to the blinded treatments. Mice were monitored for signs of distress, and humane endpoints were determined on the basis of decreased body condition. Veterinary staff monitored the mice daily and alerted researchers when a humane endpoint had been reached.

### Animals.

C57BL/6 mice were purchased from Charles River Laboratories and B6.Cg-Tcratm1Mom Tg(TcrLCMV)327Sdz (P14), and *Rag2/Il2rg-*DKO mice were purchased from Taconic. Mice were housed in the Central Animal Facility at McMaster University.

### Viruses.

LCMV-Armstrong and VSV-gp33 viruses were described previously (63–65).

### Cell lines and tumor challenge.

B16-gp33 and MC38-gp33 cell lines were generated as described previously (66). All cells were maintained at 37°C in a humidified atmosphere containing 5% CO_2_ and cultured in MEM/F11 medium containing 10% FBS, 2 mM l-glutamine, 5 mL sodium pyruvate, 5 mL nonessential amino acids, 5 mL vitamin solution (Thermo Fisher Scientific), 55 μM 2-mercaptoethanol (MilliporeSigma), 100 U/mL penicillin, and 100 ng/mL streptomycin. Naive C57BL/6 mice were challenged intradermally with 10^5^ B16-gp33 or MC38-gp33 cells in 30 μL PBS. Cured mice were rechallenged with 10^5^ MC38/MCA102/MCA102-p15E cells (67, 68) in 30 μL PBS. Tumor growth was monitored as previously described (69).

### Adoptive T cell transfer.

Spleens were collected from LCMV-Armstrong–infected mice (>1 month infected), and a single-cell suspension was prepared. LCMV GP_33–41_–specific Tmem cells were enumerated by staining with H-2D^b^-GP33 tetramer (Baylor College of Medicine), CD127 (SB/199, BD Biosciences), and CD62L (MEL-14, BD Biosciences). LCMV GP_33–41_–specific Tmem cells (10^4^ cells) in 200 μL PBS were adoptively transferred into tumor-challenged mice by i.v. injection. Twenty-four hours after adoptive T cell transfer, mice were injected i.v. with 2 × 10^8^ PFU VSV-gp33 in 200 μL PBS. Concomitantly, MS-275 (MilliporeSigma) was i.p. injected into mice (100 μg/mouse in 50 μL PBS) daily for 5 days. Selective lymphocyte depletion was conducted using mAbs (Bio X Cell) specific for CD8, CD4, or NK1.1. Mice were injected with 250 μg mAb in 500 μL PBS on day –1 and day 1 after vaccination and every 2 weeks thereafter (150 μg).

### Detection of antigen-specific responses.

Five days after vaccination, PBMCs were stimulated for 5 hours with LCMV GP_33–41_ peptide in the presence of brefeldin A (GolgiPlug, BD Biosciences). Following surface staining for CD8α (BD Biosciences), cells were fixed and permeabilized with Cytofix/Cytoperm (BD Biosciences) and stained for intracellular IFN-γ (XMG1.2, BD Biosciences). Data were acquired using an LSRFortessa flow cytometer with FACSDiva software (BD Biosciences) and analyzed with FlowJo X, version 10.0.7 (Tree Star).

### Tumor RNA extraction.

B16-gp33 tumors were excised 5 days after vaccination and snap-frozen in liquid nitrogen, and samples were then homogenized in TRIzol (Invitrogen, Thermo Fisher Scientific). RNA was extracted and purified using an RNeasy Mini kit (QIAGEN) and treated with Ambion’s DNA-free kit.

### Gene expression microarray.

Samples were profiled using Illumina MouseRef8v2 arrays (GEO GSE179337). The obtained data were processed with variance-stabilizing transformation (VST) and quantile normalization (lumi package, Bioconductor) (70). Only annotated and detected genes were selected, yielding a list of 13,088 genes for further analyses. Time-course analysis using the limma package (Bioconductor)) (71) was performed with the following contrasts: [ACT+MS-275 (day 5) – ACT+MS-275 (day 1)] — [ACT only (day 5) – ACT only (day 1)] and [ACT+MS-275 (day 3) – ACT+MS-275 (day 1)] — [ACT only (day 3) – ACT only (day 1)]. Obtained *P* values were corrected with Benjamini-Hochberg correction for multiple testing (72); corrected values of less than 0.05 were considered significant. All significant genes from the comparisons were used for PPI network construction using the Reactome FI plugin (73) in the Cytoscape environment (74). Next, modules of nodes in the network were defined and analyzed for pathway enrichment and GO biological process overrepresentation. The obtained lists of significant pathways and biological processes were used to categorize and label the modules.

GSEA (75) was performed using the whole gene expression profiles to examine the following comparisons: (a) ACT+MS-275 (day 1) – ACT only (day 1); (b) ACT+MS-275 (day 3) – ACT only (day 3); and (c) ACT+MS-275 (day 5) – ACT only (day 5). We performed the analysis using 3 gene sets: C2v4 and C7v4 from MSigDB (https://www.gsea-msigdb.org/gsea/msigdb/index.jsp) and a custom gene set (Supplemental Table 1). FDR-corrected *P* values of less than 0.05 were considered significant. Next, the results obtained for C2v4 and C7v4 were visualized using the Enrichment Map plugin (76) in the Cytoscape environment.

### Purification of tumor-infiltrating DCs.

B16-gp33 tumors were excised 5 days after vaccination and digested in 0.5 mg/mL collagenase type IV (Gibco, Thermo Fisher Scientific) and 0.2 mg/mL DNase (Roche) prepared in complete RPMI (Gibco, Thermo Fisher Scientific, 10 mL/250 mg tumor), followed by incubation at 37°C for 30 minutes as previously described (77). The digested material was CD45.2- or CD11c-enriched through magnetic selection with an EasySep Mouse Biotin or CD11c Positive Selection Kit (STEMCELL Technologies).

### Histology.

B16-gp33 tumors excised 5 days after vaccination were snap-frozen before cryostat sectioning, and the sections were then mounted on gelatin-coated histological slides. The slides were fixed in 4% paraformaldehyde for 20 minutes at room temperature and blocked with PBS containing 5% goat serum and 0.3% Triton X-100 for 30 minutes. Slides were pretreated with Leica Bond Epitope Retrieval Buffer no. 2 (Leica Biosystems) for 20 minutes before staining using H&E, IHC, or immunofluorescence (IF). For IHC staining, anti-CD8 antibody (4B11, 1:1,000, Thermo Fisher Scientific) was added and incubated overnight at 4°C. Color was developed using the Leica Bond Polymer Refine Detection Kit (Leica Biosystems) and using rabbit anti-rat antibody (1:100, Vector Laboratories). For immunofluorescence staining, anti–cleaved caspase 3 antibody (Asp175, 1:400, Cell Signaling Technology) or anti-FoxP3 antibody (FJK-16s, 1:400, Thermo Fisher Scientific) was added and incubated overnight at 4°C. Slides were incubated for 1 hour with biotinylated goat anti–rabbit IgG (Vector Laboratories) followed by Alexa Fluor 488–conjugated streptavidin (Thermo Fisher Scientific).

### Proliferation assay.

Tumor-infiltrating DCs were isolated, pulsed with LCMV GP_33–41_ peptide, and cocultured for 3 days with CFSE-labeled P14 T cells as previously reported (78). Proliferation was evaluated using several metrics including the division index (79).

### Cytotoxicity assay.

For in vitro experiments, B16-F10 cells (1 × 10^4^/well) were cocultured with tumor-infiltrating myeloid cells (1 × 10^5^/well) in 96-well, flat-bottomed microtiter plates (Corning Inc.) for 12 hours before killing was assessed. Nonadherent cells were washed using warm PBS, and 500 μg/mL MTT (Thermo Fisher Scientific) solution was added before incubating the plates for 4 hours. Solubilization of the formazan byproduct was done by aspirating the MTT solution and adding DMSO. The absorbance was measured at 540 nm using a Synergy microplate reader (BioTek). For in vivo experiments, target bulk splenocytes were isolated and pulsed with 1 μg/mL LCMV GP_33–41_ peptide for 1 hour and labeled with CFSE at a final concentration of 5 μM/mL (CFSE^hi^) in RPMI media 1640 with 2% FBS for 15 minutes as previously described (80). The cells were mixed at a 1:1 ratio with unpulsed bulk splenocytes labeled with 0.5 μM/mL CFSE (CFSE^lo^) and infused i.v. into treated mice 4 days after treatment. After 24 hours, spleens were harvested and processed for flow cytometric detection of CFSE-expressing cells.

### Statistics.

GraphPad Prism (GraphPad Software) was used for graphing and statistical analyses. An unpaired, 2-tailed Student’s *t* test and ANOVA were used to query immune response data. A log-rank test was used to assess survival data. All data are presented as the mean ± SEM, and differences between means were considered significant at a *P* value of less than 0.05. Error bars indicate 95% CIs throughout.

### Study approval.

All animal studies complied with the Canadian Council on Animal Care guidelines and were approved by McMaster University’s Animal Research Ethics Board (Hamilton, Ontario, Canada).

### Data and materials availability.

The expression data reported in this study are available in the NCBI’s GEO database (GEO GSE179337).

## Author contributions

AN designed and conducted the majority of the experiments described and wrote the manuscript. LH prepared the samples for microarray analysis and qRT-PCR. RH constructed the MCA102-p15E cell line. LC helped monitor the therapeutic efficacy of ACT+MS-275 in the gp33 model. SRW provided guidance during manuscript preparation. YW supervised the study and participated in the conception of the experimental designs.

## Supplementary Material

Supplemental data

Supplemental table 1

## Version 1 08/16/2022

In-Press Preview

## Version 2 10/03/2022

Electronic publication
