# Supplementary material for: HDACi promotes inflammatory remodeling of the tumor microenvironment to enhance epitope spreading and antitumor immunity
Source: J Clin Invest. 2022 Oct 3;132(19):e159283. doi: 10.1172/JCI159283 (PMC9525113; doi:10.1172/JCI159283)
Supplement: Supplemental data [file jci-132-159283-s100.pdf]

Supplementary Figure 1

A

Differentially expressed genes (limma, decideTests, BH correction)

Regulation	↑			↓		
Absolute Fold Change	All	>1.5	>2.0	All	>1.5	>2.0
3d vs. 1d	61	24	3	19	7	2
5d vs. 3d	327	99	9	321	110	34
5d vs. 1d	1262	299	24	1147	411	160

Days post-treatment

cMap1 (5d vs. 1d: 299, 411)

cMap2 (5d vs. 1d: 24, 160)

B

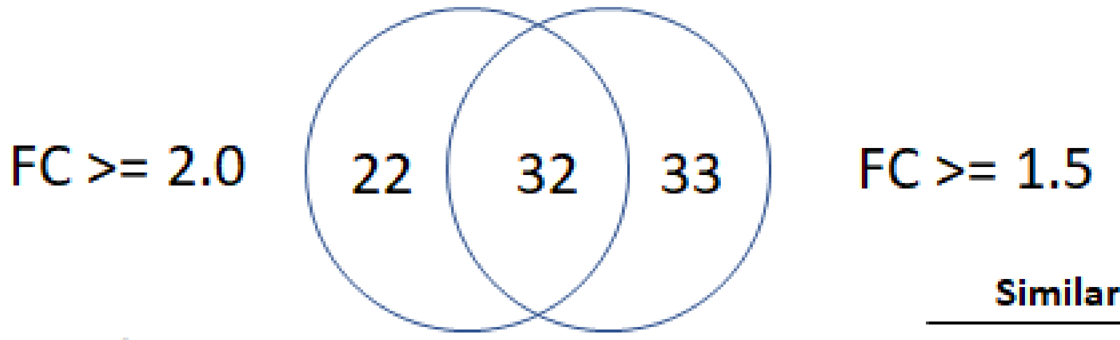

C

Compound		Similarity FC2.0	Similarity FC1.5
MS-275	HDAC inhibitor	-0.929	-0.782
DL-thiorphan	membrane metalloendopeptidase inhibitor	-0.89	-0.722
quinostatin	PI3K inhibitor	-0.807	-0.87
rottlerin	Enzyme inhibitor	-0.793	-0.729
0297417-0002B		-0.75	-0.829
alexidine		-0.717	-0.573
puromycin	antibiotics	-0.709	-0.65
pyrvinium	antihelmintic	-0.672	-0.717
propafenone	Sodium-channel blocker	-0.665	-0.509
etacrynic acid	Enzyme inhibitor	-0.66	-0.709
ivermectin	Activates chloride channels	-0.658	-0.662
phthalylsulfathiazole		-0.624	-0.593
medrysone	corticosteroid	-0.616	-0.597
methylergometrine	oxytocics	-0.58	-0.644
dequalinium chloride	Disinfectant	-0.567	-0.533
thioridazine	Dopamine antagonist	-0.563	-0.649
levonorgestrel	Synthetic progesterone	-0.558	-0.56
withaferin A		-0.541	-0.61
dipyridamole	phosphodiesterase inhibitor	-0.535	-0.549
trichostatin A	HDAC inhibitor	-0.535	-0.653
flunisolide	corticosteroid	-0.514	-0.487
vorinostat	HDAC inhibitor	-0.512	-0.556
pimozide	Dopamine blocker	-0.51	-0.58
LY-294002	Enzyme inhibitor	-0.507	-0.613
0175029-0000		-0.498	-0.634
chlorpromazine	Dopamine antagonist	-0.458	-0.441
tretinoin	ATRA, retinoic acid	-0.45	-0.427
trifluoperazine	Dopamine antagonist	-0.439	-0.653
sirolimus	Immunosupressive agent	-0.323	-0.615
wortmannin	Immunosupressive agent; PI3K inhibitor	-0.3	-0.576
15-delta prostaglandin J2		-0.296	-0.387
valproic acid	HDAC inhibitor	-0.206	-0.307

Figure S1, related to Figure 2: Connectivity mapping of differentially expressed (DE) genes (A) DE genes in ACT+MS-275 relative to ACT only treatment at 1, 3, and 5 days post-treatment. The Day 5 vs. Day 1 comparison was used to create two cMap signatures: genes with fold change of at least 1.5 (cMap1), and genes with fold change of at least 2.0 (cMap2). Both signatures were used to query the Connectivity Map database and compared to chemical perturbation gene signatures. **(B)** Venn diagram of compounds that show significant signature overlap **(C)** Ranking of the 32 overlapping compounds based on similarity to the FC >= 2.0 signature

Supplementary Table 1

Table S1.csv

Supplementary Table 2

Day 1 GSEA - positively enriched in ACT+MS-275 vs ACT only

NAME	SIZE	FDR q-val	Leading Edge
ADAPTIVEIMMUNITY_DOWN	25	0.001	TLR4, CTLA4, RORC, STAT3, IFNG, TGFB1, SLC11A1, IL18, CXCL10, CCR5, C3
ADAPTIVEIMMUNITY_UP	18	0.001	ICAM1, IFNG, CD83, CD40, PRF1, CD86, CD68, SLC11A1, IL18
ANTIVIRALRESPONSE_UP	74	0.000	MX1, IRAK4, TLR13, AZI2, RELA, IFIH1, TYK2, IFNAR2, CASP8, IFITM2, TLR4, DDX3X, TBK1, RIPK1, DDX58, MAPK3, MAP3K1, MAP2K1, IL15, TLR2, CD40, TLR3, MYD88, TLR7, PSTPIP1, CD86, SPP1, TLR13, CTSB, IRF5, STAT1, CTSS, IL18, CXCL10, CASP1, IFIT2
IFNS_AND_RECEPTORS_DOWN	48	0.000	IL15, IL7R, IFNG, EBI3, IL12RB1, IL28RA, IL13RA1, IRF2, IL10RB, IFIT3, IRF8, IRF5, IRF1, CXCL10, IFIT2
INFLAMMASOMES_DOWN	46	0.001	MAPK3, IL33, IFNG, BIRC2, MAPK13, NLRX1, MYD88, CCL7, TXNIP, IL18, IRF1, CIITA, NOD1, CASP1
INFLAMMASOMES_UP	9	0.048	BCL2L1, HSP90B1, PSTPIP1, CTSB
INFLAMMATORYCYT_CHE_DOWN	43	0.000	IL33, IL15, CSF1, IFNG, CCL2, IL10RA, CCL3, CXCL13, CXCL16, CXCL9, IL10RB, CCL6, CCL7, TNFRSF11B, CCL9, CXCL10, CCR5
INFLAMMATORYRES	49	0.000	IL1RN, CCL25, FASL, RIPK2, BCL6, TLR4, CCL11, LY96, CSF1, IFNG, TLR2, CCL2, CD40, TLR3, MYD88, CCL3, TLR7, CXCL9, IL10RB, CCL7, C4B, CEBPB, IL18, CXCL10, C3
MELANOMA_UP	18	0.018	ENC1, TRP53INP1, RB1, SOCS2, SOCS3, IRF8

Day 5 GSEA - negatively enriched in ACT+MS-275 vs ACT only

NAME	SIZE	FDR q-val	Leading Edge
INFLAMMATORYRESPONSE_AUTOIMMUNITY_DOWN	49	0.000	CCL5, IL1B, CD14, IL1RN, CD40, CCL7, C4B, IL10RB, TLR2, C3, TNF, IL18, NOS2, TLR7, CCL2, BCL6, CEBPB, IL1A, LTB, FASL, ITGB2, CXCR4, NFKB1, CXCL9
INFLAMMATORYCYT_CHE_DOWN	43	0.002	CXCL16, CCR5, CCL6, TNFRSF11B, CCL5, IL1B, IL1RN, CCL7, IL10RB, IL15, IL10RA, TNF, IL2RG, CCL9, CCL2, IL1A, LTB, FASL, CXCL9, IL33, CXCL12, OSM, IL16
ANTIVIRALRESPONSE_UP	74	0.008	CASP1, CCL5, CTSS, TLR13, IL1B, CD14, CD86, CD40, TLR2, IL15, IRF5, TNF, IL18, TLR7, NFKBIA, IL1A, ATG12, NFKB1, AZI2, CTSB
ADAPTIVEIMMUNITY_UP	18	0.008	CD68, SLC11A1, CD86, CD40, CD83, IL18, ICAM1
ADAPTIVEIMMUNITY_DOWN	25	0.016	CCR5, SLC11A1, CCL5, C3, TNF, IL18, NOS2, PDCD1LG2, TGFB1, TLR4, STAT4, TLR6

Table S2, related to Figure 2A: Enriched custom gene sets after gene set enrichment analysis (GSEA)

Supplementary Figure 2

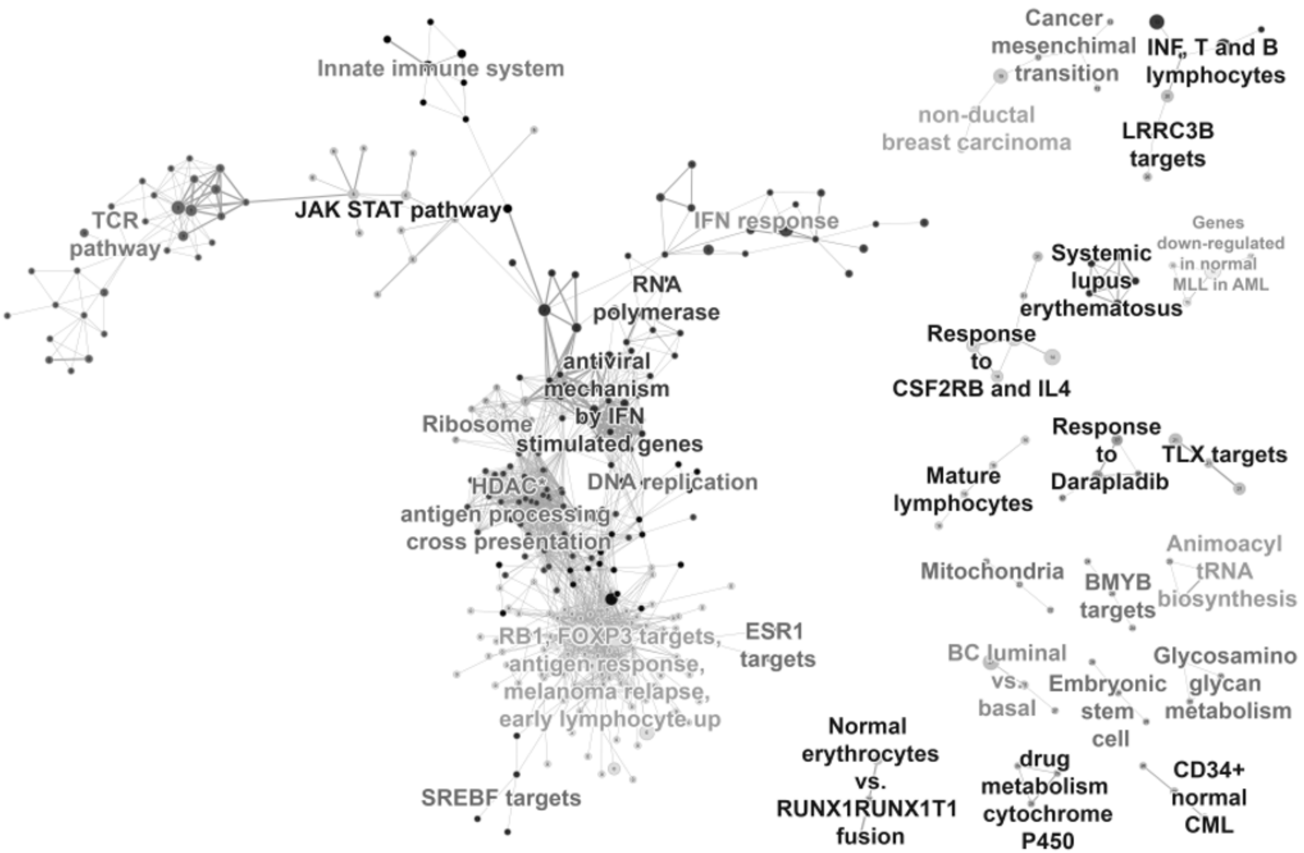

Figure S2, related to Figure 2C: Fully-annotated curated gene sets (C2) enrichment map

Enrichment map with full annotations for every gene set cluster.

Supplementary Table 3

Module	Nodes in Module	Genes
0	28	ANAPC4, ANAPC5, FZR1, GSPT2, HECTD3, MGRN1, PARD6A, PJA1, PSMB4, RBCK1, RNF4, RPL21, RPL23, RPL30, RPL38, RPS10, RPS13, RPS19, RPS27A, RPS6, RSL1D1, SKP2, SPSB1, SRP14, TRIP12, UBA2, UBE2L3, UBE2S
1	22	ALYREF, CBFB, CCAR1, CD164, EFTUD2, HNRNPA2B1, HNRNPM, NCBP1, PABPN1, PHF5A, PTBP1, RALY, RBM4, RBM8A, RBMX, SF3B2, SRSF1, SRSF2, SRSF3, SRSF6, THOC5, TRA2A
2	22	ASF1B, BCL11B, CHD4, CTSH, DNMT1, DNMT3B, H2AFJ, H2AFY, HDAC11, HDAC2, HIRA, HIST1H2AH, HIST2H2AB, IKZF1, KLF7, NAP1L1, SALL4, SMARCA2, SMARCE1, SUV39H1, TCF19, UHRF1
3	20	ALPL, ASAH2, BZW2, CHMP1B, CRABP2, CTF1, DBP, DRAM2, EP300, FABP3, HCN2, ILF2, INSIG2, MFGE8, NDRG2, PSTPIP1, PTGDS, VAMP5, WFDC1, ZHX2
4	19	CSTF1, DDX20, GEMIN5, GEMIN6, HNRNPK, LSM10, LSM2, LSM4, LSM6, PRPF19, SF3A3, SF3B3, SMN1, SNRNP70, SNRPA, SNRPB, SNRPD2, SNRPD3, TXNL4A
5	17	APITD1, AURKA, AZI1, BUB1B, CENPM, CENPN, CLIP1, KIF2C, MAD1L1, NDC80, NDE1, NUDC, PLK1, PMF1, SGOL1, SPC24, ZW10
6	17	ABR, ARFIP2, ARHGAP17, ARHGAP25, ARHGDIG, ARHGEF3, CDC42EP2, CTTN, DEPDC1B, HAP1, MTSS1, NCKAP1L, PLEK, RAC2, RHOBTB2, RHOF, SELPLG
7	15	DCPS, ERCC1, ERCC2, GTF2E2, GTF2H3, NFIB, NFIX, POLR2D, POLR2E, POLR2F, POLR2H, POLR2J, RNMT, WHSC2, ZFP30
8	14	CXCL16, CYSLTR1, FPR2, GNA15, GNAS, GNG2, GNGT2, GPR18, GPR65, GPR68, GRK6, P2RY14, P2RY6, PLCL2
9	12	CDC45, CDC7, CDT1, GINS4, MCM10, MCM2, MCM3, MCM4, MCM5, MCM6, MCM7, ORC6
10	12	CDC25A, CDK1, FOXM1, LMNB1, MELK, NCAPH2, PBK, PKMYT1, PRC1, SPAG5, TUBG1, UCK2
11	11	AAAS, BANF1, MCFD2, NUP133, NUP210, NUP43, NUP50, NUP85, NUP93, RANGAP1, SNUPN
12	11	EARS2, MVD, NVL, PSMA7, PSMB1, PSMC2, PSMC5, PSMD13, PSMD4, PSMD7, RAD23A
13	10	ATRIP, CINP, CLSPN, MSH6, POLE, RAD1, RFC1, RFC5, RPA1, TIMELESS
14	9	CD28, CD3E, DBNL, LCK, LIME1, PDCD1LG2, PTPN22, RGS1, SLA
15	9	APBB1IP, ELMO1, RAP1B, RAPGEF1, RAPGEF5, RASGRP1, RGL1, RIT1, RND3
16	8	CNOT2, DDX49, EIF2S2, EIF3G, EIF4A1, EIF4E3, EIF4G1, RQCD1
17	8	NFKB2, NFRKB, NR1H3, RELA, RELB, S100B, TSC22D3, ZDHHC13
18	8	ACTR5, LEO1, MORF4L1, NHP2, PAF1, RUVBL2, TFPT, WRAP53
19	7	ACOX3, FADS2, LMO2, MED15, MED18, MED8, RXRA
20	7	ACTR1B, DCTN4, KIF22, KIF3C, LAMP2, RILP, STX12
21	7	CCT3, CCT6A, CCT8, FBXL5, FBXW4, PFDN2, PFDN4
22	7	ALS2, ARL8B, RAB11B, RAB5A, RAB5B, RABEP1, RIN2

Table S3, related to Figure 2D: Modules derived from the network of differentially expressed genes

Supplementary Figure 3

A

Differentially expressed genes (limma, decideTests, BH correction)

Regulation	↑			↓		
Absolute Fold Change	All	>1.5	>2.0	All	>1.5	>2.0
3d vs. 1d	61	24	3	19	7	2
5d vs. 3d	327	99	9	321	110	34
5d vs. 1d	1262	299	24	1147	411	160

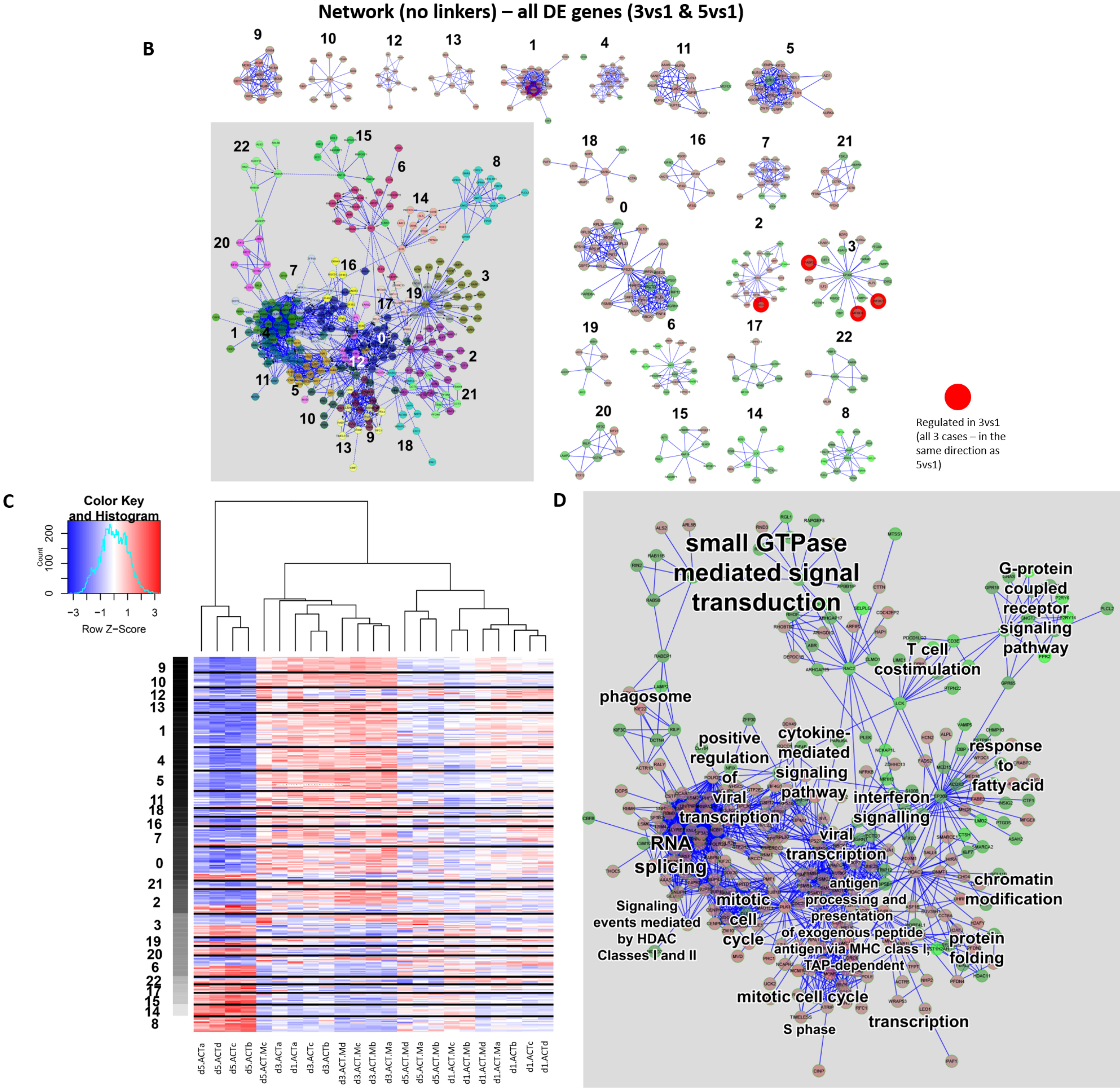

Figure S3, related to Figure 2D: Gene module ranking based on MS-275 up-regulation
(A) Differential gene expression comparison of ACT+MS-275 treatment vs ACT alone from Day 1 to Day 5 (FDR p<0.05). **(B)** Protein-protein interaction network where clusters of differentially expressed genes (DE) were assembled into gene modules based on D3 vs D1 and D5 vs D1. **(C)** Expression heatmap and sorting of modules based on percentage of genes up-regulated by MS-275 for D3 vs D1 and D5 vs D1 comparisons **(D)** GO term assignment to each module and color assignment based on relative up-regulation (red) or down-regulation (green) by MS-275.

Supplementary Figure 4

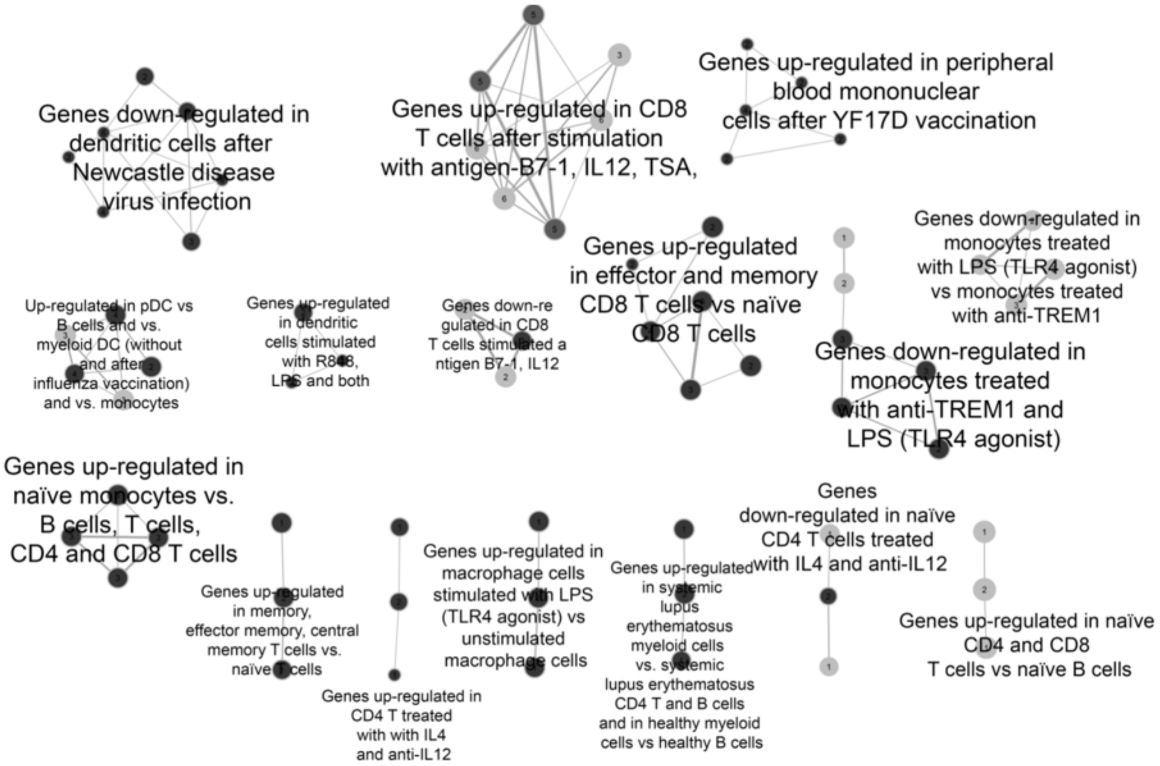

Figure S4, related to Figure 2F: Fully-annotated immunologic signatures (C7) enrichment map
Enrichment map with full annotations for every gene set cluster.
